# Echocardiography as a guide for fluid management

**DOI:** 10.1186/s13054-016-1407-1

**Published:** 2016-09-04

**Authors:** John H. Boyd, Demetrios Sirounis, Julien Maizel, Michel Slama

**Affiliations:** 1 Critical Care Research Laboratories, Centre for Heart Lung Innovation at St. Paul’s Hospital University of British Columbia, 1081 Burrard Street, Vancouver, BC V6Z 1Y6 Canada; 2 Department of Critical Care Medicine, University of British Columbia, Vancouver, BC Canada; 3 Faculty of Medicine, University of British Columbia, Vancouver, BC Canada; 4 Réanimation médicale, CHU Sud, Amiens, France; 5 Unité INSERM 1088, UPJV, Amiens, France

**Keywords:** Shock, Point-of-care ultrasound, Echocardiography, Resuscitation

## Abstract

**Background:**

In critically ill patients at risk for organ failure, the administration of intravenous fluids has equal chances of resulting in benefit or harm. While the intent of intravenous fluid is to increase cardiac output and oxygen delivery, unwelcome results in those patients who do not increase their cardiac output are tissue edema, hypoxemia, and excess mortality. Here we briefly review bedside methods to assess fluid responsiveness, focusing upon the strengths and pitfalls of echocardiography in spontaneously breathing mechanically ventilated patients as a means to guide fluid management. We also provide new data to help clinicians anticipate bedside echocardiography findings in vasopressor-dependent, volume-resuscitated patients.

**Objective:**

To review bedside ultrasound as a method to judge whether additional intravenous fluid will increase cardiac output. Special emphasis is placed on the respiratory effort of the patient.

**Conclusions:**

Point-of-care echocardiography has the unique ability to screen for unexpected structural findings while providing a quantifiable probability of a patient’s cardiovascular response to fluids. Measuring changes in stroke volume in response to either passive leg raising or changes in thoracic pressure during controlled mechanical ventilation offer good performance characteristics but may be limited by operator skill, arrhythmia, and open lung ventilation strategies. Measuring changes in vena caval diameter induced by controlled mechanical ventilation demands less training of the operator and performs well during arrythmia. In modern delivery of critical care, however, most patients are nursed awake, even during mechanical ventilation. In patients making respiratory efforts we suggest that ventilator settings must be standardized before assessing this promising technology as a guide for fluid management.

## Background

A routine but challenging task facing acute care physicians is to identify and treat patients at risk for acute organ failure as a result of inadequate systemic perfusion and oxygen delivery. The decision to administer supplemental intravenous fluids to the patient at risk is built upon the belief that additional volume expansion will, or will not, increase cardiac output. Although there are many unknowns in a critically ill patient, fundamental cardiovascular physiology represents a touchstone from which decisions can be made.

One important premise is that during the time it takes to administer bolus intravenous fluid, one can assume a constant cardiac contractility. Changes in stroke volume in response to intravenous fluids are therefore mainly determined by changes in ventricular end-diastolic volume. At constant cardiac contractility, the relationship between stroke volume and ventricular end-diastolic volume has been classically described by Patterson and Starling [1], Sarnoff and colleagues [2–5], and Guyton and Coleman [6]. At low ventricular end-diastolic volume, the stroke volume increases briskly with administration of intravenous fluids. This immediately increases both cardiac output and oxygen delivery. Unfortunately, once the patient reaches their plateau ventricular end-diastolic volume, further increasing preload with intravenous fluids will not improve cardiac output and has been shown to result in clinical harm [7–13]. It is therefore critical that the clinician uses the best means at their disposal to judge whether additional intravenous fluids will result in benefit (increased cardiac output, oxygen delivery, and ultimate reversal of impending organ failure) [14] or harm (tissue edema, hypoxemia, and excess mortality). While the clinical effect of fluids in a given patient will be determined through careful examination and a number of parameters such as reduction in lactate, urine output, and level of consciousness, in the following sections when we refer to the presence of “fluid responsiveness” we refer to a measurable increase of 15 % in cardiac output.

## Is the patient on the steep portion of the Starling curve?

Clinical prediction of fluid responsiveness was first studied using single measurements of cardiac filling volumes (preload). These include the direct measurement of right atrial pressure as a surrogate of volume, also referred to as central venous pressure (CVP) and less commonly as the pulmonary capillary wedge pressure, which in ideal situations is synonymous with left atrial pressure as a surrogate of left ventricular end-diastolic volume. As has been extensively reviewed elsewhere, static measures of preload perform no better than chance in patients who are critically ill. It is now recognized that bedside maneuvers which rapidly change preload are more discriminative than static measures. Currently, passive leg raising (PLR) and respiratory variation in thoracic pressure are the two techniques used to vary preload. Within 1 minute of bilateral PLR, there is an effective increase in preload through recruitment of blood pooled in the legs [15]. In the patient not on vasopressors, an increase in blood pressure suggests that the patient will respond to a fluid bolus, whereas in those on vasoactive medications there is no detectable change in blood pressure and the output of interest is change in cardiac output. This technique is therefore best suited to patients who are not yet on vasopressors, with normal intrathoracic pressures (mechanically ventilated patients often fail to augment their preload as definitely), and those without significant abdominal pathology.

Variation in intrathoracic pressure during tidal breathing is the other cause of varied right or left cardiac preload. It is important to recognize that the type of respiration (spontaneous versus controlled) will determine the resultant physiology. In mechanically ventilated patients with no respiratory effort, the positive inspiratory pressure transfers blood from the lungs to the left heart, resulting in an increase in stroke volume. This is seen as a rapid increase in pulse pressure. At high levels of positive intrathoracic pressure one can also decrease the venous return, and after transit of this reduced blood volume from the right to left heart there can be a decline in pulse pressure. The larger the fluctuations in pulse pressure as a result of respiration, the greater the chance that the patient will increase cardiac output in response to fluid. This method cannot be used during arrhythmias such as atrial fibrillation or with very rapid heart rates.

Respiratory changes in intrathoracic pressure also occur in the dimensions of both the inferior and superior vena cavae. These changes also depend on the degree of intrathoracic pressure change and on the compliance of the vena cavae. Positive intrathoracic pressure increases the size of the inferior vena cava (IVC), while negative intrathoracic breaths reduce its size. When the vena cava is distended, the compliance markedly reduces. A large, nonfluctuant IVC therefore suggests that the patient is not on the steep (volume responsive) portion of the Starling curve. The IVC diameter is easily and reproducibly measured 1–2 cm from the right atrial junction using transthoracic ultrasound [16–21].

## The patient following initial resuscitation

Venous return, central venous pressure, and cardiac output are tightly coregulated as described by Patterson and Starling [1], Sarnoff and colleagues [2–5], and Guyton and Coleman [6]. Highly predictable under normal circumstances, the ability to increase cardiac output through augmentations in venous return and central venous pressure changes dramatically in the patient who develops shock refractory to intravenous fluids. In 2016, standardized protocols dictate that following the recognition of nonhemorrhagic shock the patient will rapidly receive at least 20 ml/kg fluid [22]. Fifty percent of patients [23] do not achieve adequate perfusion with modest volume expansion and thus require vasopressors to maintain circulatory tone. In these patients, intravenous volume expansion during the first 6–12 hours following admission increases dramatically to 50–70 ml/kg [23, 24]. Norepinephrine, the vasopressor of choice in most circumstances [25], will not only increase arterial blood pressure through increased systemic vascular resistance but has a significant effect upon capacitance vessels, both arterial and venous, resulting in effective fluid loading to the right heart [26]. It is evident that infusion of an entire blood volume of new fluid into the circulation, along with recruitment of circulatory capacitance, results in an extreme change to venous return, central venous pressure, and cardiac output.

In addition to the direct influence of the cardiovascular system, cardiopulmonary interactions play a crucial role in establishing the equilibrium of venous return, central venous pressure, and cardiac output. This is because 70–90 % of patients with shock require mechanical ventilation [25, 27]. In the early phase of resuscitation during which patients are sedated to facilitate investigations and treatment, mean airway pressures of up to 24 cmH_2_O/18 mmHg [28] have significant but unpredictable effects. These high thoracic pressures cause a decline in venous return and/or a functional unloading of the left ventricle through pressurization of the heart and thoracic aorta.

## What can the clinician expect to find on clinical examination and echocardiography following initial volume resuscitation?

Central venous pressure in health is tightly governed at 0–5 mmHg [1–6], resulting in an IVC diameter of 13–21 mm when supine, and a collapse of more than 50 % upon quiet inspiration [16, 17, 19]. In patients who have received the current aggressive early volume expansion we know the newly established value for central venous pressure will be 9–15 mmHg [12, 24]. This change in venous pressure is highly influential upon the vena caval diameter as measured by transthoracic echocardiography. In our recently published study into the utility of ultrasound following volume expansion to guide therapy in nonhemorrhagic shock [14] we went back to review the impact of 30 ml/kg intravenous fluid resuscitation upon the IVC diameter and fluctuation. In 110 subjects the IVC diameter following intravenous volume expansion was increased by 35 % from normal values to 17–29 mm. Furthermore, in nearly half (45 %) of the patients there was no variation in diameter according to respiration. In a further 20 % of patients there was >0 but <15 % variability (the median cutoff point of IVC collapse which defines fluid responsiveness). This information may be of utility for the clinician without access to ultrasound, who may choose to restrict further fluids based upon a pretest probability of 0.65 that their fluid-resuscitated patient on vasopressors will not augment cardiac output as a result of further fluids.

## Summary of what the clinician can expect when using central venous pressure and ultrasound to guide fluid therapy in the ventilated critically ill patient following resuscitation for shock

1. A central venous pressure of 9–15 mmHg.
2. Maximum IVC diameter of 17–29 mmHg
3. According to the increase in IVC diameter upon mandated inspiration, 2/3 patients will be deemed nonresponsive to fluid.

## Clinical methods at the bedside to assess volume response

### Using ultrasound to monitor stroke volume while performing the passive leg raising maneuver

Passive leg raising (PLR) is one of the most versatile techniques to assess fluid needs in ICU patients. PLR can be performed at the bedside in both mechanically ventilated patients and in spontaneously breathing patients [15, 29–31]. Meta-analysis of 23 studies with a combined total of 1013 patients from a wide range of clinical settings demonstrated that the global predictive value of PLR was strong. The test performed very well with a pooled sensitivity of 86 %, specificity of 92 %, and a summary AUROC of 0.95 [30]. In another meta-analysis, 21 studies were analyzed and the pooled correlation between the PLR-induced versus fluid-induced increases in cardiac output was 0.76. The pooled sensitivity was 0.85, the pooled specificity was 0.91, and the pooled AUROC was 0.95 [15]. This maneuver is easy to perform at the bedside. The patient is placed in a semirecumbent position with the head of the bed 30–45° above the horizontal. The maneuver consists of rapidly moving the bed to simultaneously elevate the lower limbs to 30–45° above the horizontal while lowering the head of the bed to 0^o^ (supine). This maneuver transfers blood from the legs and the splanchnic reservoir to the intrathoracic compartment, rapidly increasing the preload, thereby testing the preload dependency of the heart. Using PLR, 250–350 ml of blood is transferred from the legs to the heart and this method is entirely reversible [30]. It is essential that this maneuver should be done from the semirecumbent position because this increases the blood shift and accentuates the change in cardiac output compared with a supine start [32]. Cardiac output changes can be detected 1–2 minutes after the PLR maneuver using echocardiography [31]. It is useful to note that there is a close correlation between the changes in cardiac output or stroke volume induced by the PLR and that achieved through equivalent intravenous volume expansion. In other words, the change is not simply a threshold effect, and the greater the increases in cardiac output and stroke volume during PLR, the greater will be the increase in these parameters after fluid infusion. Arrhythmia should have no effect on the diagnostic performance because the effect of PLR is measured over multiple heartbeats and multiple breaths, likely nullifying potential distorting effects of arrhythmia and spontaneous breathing, respectively [30], but this has yet to be confirmed by a large prospective study in this population. The PLR maneuver seems inaccurate in patients with very significant intra-abdominal hypertension, as demonstrated by Mahjoub et al. [33].

#### Advantages of the passive leg raising maneuver

1. PLR can be performed regardless of arrhythmia or mode of ventilation.
2. The PLR is not simply “positive” versus “negative”; the degree of increase in stroke volume to PLR predicts the increase in these parameters to fluids.

#### Disadvantages of the passive leg raising maneuver

1. The interobserver and intraobserver reliability of measurements in cardiac output is highly operator dependent. A skilled operator is required to achieve high-quality measures of aortic blood flow.

### Using ultrasound to monitor stroke volume while receiving controlled mechanical ventilation

Aortic flow variations during mechanical ventilation may be a superior measure of what is observed clinically as stroke volume variation (SVV), a parameter correlated with fluid responsiveness [34]. Feissel et al. [35] assessed the variation of the maximal velocity during the respiratory cycle and found that variation greater than 12 % accurately predicted fluid responsiveness of ICU patients. The aortic blood flow is recorded from an apical five-chamber view using pulsed Doppler imaging. Aortic blood flow variation shares the same limitations as pulse pressure variation. These two parameters may be used only in patients without arrhythmia and seem invalid in patients with right ventricular dilation or dysfunction [36]. The pathophysiology of these parameters is based on the effects of mechanical ventilation, which induces transpulmonary and intrathoracic pressure change. The magnitude of these effects depends mainly on the transmission of airway pressure variations to the heart. Open chest conditions therefore make all of these parameters invalid to assess fluid need. Similarly, protective mechanical ventilation (in which a low tidal volume is used to decrease the plateau pressure and driving pressure) is now widely used for ARDS patients, in whom the low tidal volume decreases airway pressure variations and may dramatically decrease the hemodynamic effects of mechanical ventilation [37]. De Baker and Scolletta [38] demonstrated that low tidal volume (<8 ml/kg) invalidates the cutoff value of 12 % for pulsed pressure variation (PPV) (a surrogate of stroke volume variation). In an attempt to solve this problem, Liu et al. [39] suggested estimating pleural pressure variations as a surrogate of thoracic pressure variations in ARDS patients and then adjusting the PPV accordingly in order to improve prediction and prevent false negatives for fluid responsiveness. This approach, however, requires measurement of esophageal pressure using a balloon catheter, increasing the complexity of care, and is therefore used clinically in a small number of centers.

#### Advantages of measuring aortic blood flow during mechanical ventilation

1. No additional maneuvers are required; standard mechanical ventilation provides the dynamic changes in preload.

#### Disadvantages of measuring aortic blood flow during mechanical ventilation

1. The interobserver and intraobserver reliability of measurements in cardiac output is highly operator dependent. A skilled operator is required to achieve high-quality measures of aortic blood flow.
2. Not accurate during arrhythmia.
3. Of limited utility with “open lung” ventilator strategies which reduce pleural pressure swings.

## Mechanical ventilation induced variations in vena-caval diameter

### Controlled ventilation

Under controlled mechanical ventilation, positive pressure is applied into the thorax. The superior vena cava (SVC) is therefore subjected to this positive pressure during mechanical insufflation. Vieillard-Baron et al. [40] demonstrated that respiratory variation of the superior vena cava analyzed using transesophageal echocardiography accurately predicts fluid responsiveness of ICU patients with a cutoff value of 36 %. Following this study there was a revolution in ultrasound technology which facilitated a less invasive approach, and in 2016 most clinicians prefer transthoracic echocardiography rather than the transesophageal approach to assess the IVC. Multiple studies analyzed the IVC in ICU patients under controlled mechanical ventilation [41–44]. Together these studies demonstrated that, like static measures of central venous pressure, the absolute size of the IVC was not able to accurately predict the effect of fluid infusion on cardiac output. In contrast, the change in IVC diameter induced by intrathoracic pressure swings during mechanical ventilation is useful. Using the ratio between maximal size minus minimum size to the average of these two values, Feissel et al. [43] found that a variation higher than 12 % was associated with an increase of cardiac output after fluid infusion. Barbier et al. [41] found that 18 % was the cutoff value by using the ratio of the maximal size minus the minimum size to the minimum size. All of these measurements were made on M-mode images of a longitudinal view of the IVC obtained from a subcostal window. Intra-abdominal hypertension, the tidal volume, and the patient’s inspiratory efforts in spontaneous breathing may be possible limitations of this approach [45].

### Spontaneously breathing patients

**Fig. 1:**
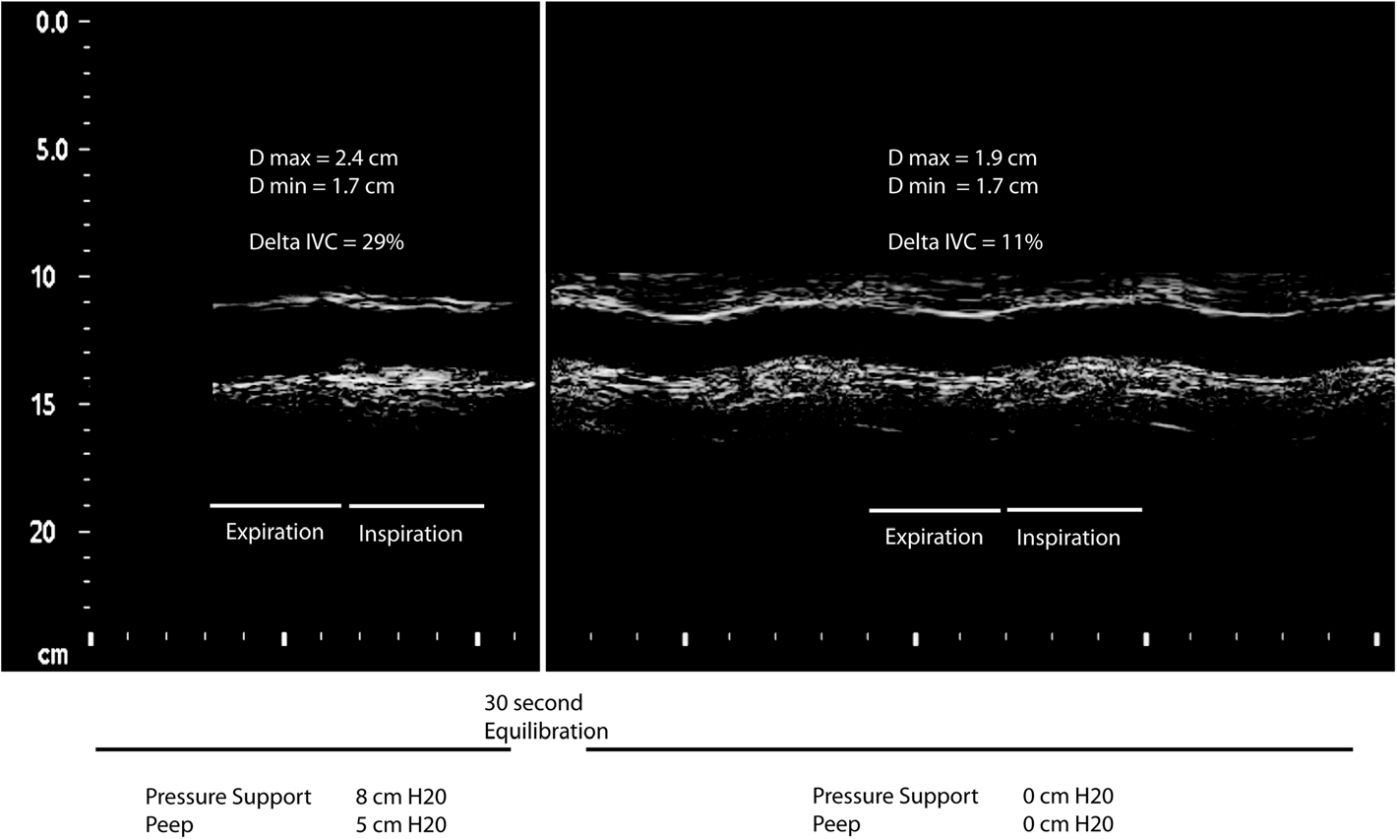
A 57-year-old male patient admitted with septic shock 18 hours before imaging required 0.2 μg/kg/minute of norepinephrine to maintain a mean arterial blood pressure of 70 mmHg. Central venous pressure via the right internal jugular catheter was 13 mmHg and he was in atrial fibrillation, rate of 100 beats/minute. Sedation had been discontinued and the patient was awake and spontaneously breathing on a mechanical ventilator. Using a subcostal approach the IVC was imaged using M-mode at 1.5 cm from the IVC–right atrial junction. The patient then began a spontaneous breathing trial, with some translational movement of the IVC noted, and imaging continued. In this case the IVC diameter during inspiration did not change according to the level of pressure support, whereas the end-expiratory IVC diameters were markedly greater with positive pressure applied. Thus the delta IVC during usual mechanical ventilation was 29 %, while during his spontaneous breathing trial the delta IVC was only 11 %. A CardioQ™ esophageal Doppler probe was in place and an optimal descending aortic blood flow was calculated. In this patient the stroke volume increased from 49 to 65 ml (33 % increase) with a 500 ml bolus of plasmalyte™, and thus was truly volume responsive. *IVC* inferior vena cava, *PEEP* positive end-expiratory pressure

Following the initial resuscitation, most patients in the modern era are nursed while awake and are encouraged to breathe in collaboration with the ventilator. This means that in awake, spontaneously breathing patients the swings in pleural pressures during inspiration which are transmitted to the IVC can vary from deeply negative (in those ventilated on CPAP only, as in a spontaneous breathing trial) to neutral/positive in cases with high levels of pressure support or neuromuscular weakness. It has recently been shown in healthy volunteers that the change in IVC diameter is highly correlated with respiratory effort [46]. In our center we have found that the degree of additional pressure support applied in a spontaneously breathing patient on mechanical ventilation will dramatically change both the IVC diameter and the degree of IVC collapse we observe. Figure 1 shows IVC tracings in a patient who was first scanned while they were assisted with pressure support of 8 cmH_2_O above PEEP. Immediately following this scan the patient began a spontaneous breathing trial with 0 cmH_2_O additional support. Clearly both the IVC diameter and fluctuation are highly influenced even at modest levels of pressure support. This intuitive but under-recognized fact has important implications when interpreting the results of an ultrasound examination in awake patients with the intent of guiding fluid therapy. Reports of changes in IVC diameter in spontaneously breathing patients have found that these failed to accurately predict fluid responsiveness. For instance, IVC respiratory variations >42 % in spontaneously breathing patients demonstrated a high specificity (97 %) and a positive predictive value (90 %) to predict an increase in CO after fluid infusion with a cutoff value >42 % [47] but a low sensitivity and negative predictive value. A recently published physiology-based opinion suggests that IVC respiratory variations are in fact prone to both false negatives and positives due to five major categories: ventilator settings, patient’s inspiratory efforts, lung hyperinflation, cardiac conditions impeding venous return, and high intra-abdominal pressures [45].

#### Advantages of measuring vena caval diameter as a surrogate of volume responsiveness during mechanical ventilation

1. No additional maneuvers are required; the standard mechanical ventilation provides the dynamic changes in preload.

#### Disadvantages of measuring vena caval diameter as a surrogate of volume responsiveness during mechanical ventilation

1. Of limited utility in controlled modes of ventilation using “open lung” strategies which reduce pleural pressure swings.
2. In awake patients, the IVC diameter and collapse are highly dependent upon the patient’s respiratory effort and levels of ventilatory support.
3. While echocardiography is of great value in the diagnosis of right ventricular failure, more direct measures of left ventricular performance such as descending aortic Doppler flow are required in this population.

## Future directions

### Left ventricular outflow tract obstruction

In a recent study, Chauvet et al. [48] found that 22 % patients in the early phase of septic shock presented with functional left ventricular outflow tract obstruction (LVOTO). In most of these patients, fluid infusion decreased this obstruction, increased cardiac output, and clinically improved the patient [48]. Pending confirmation and prospective validation, LVOTO may be considered a new fluid-responsiveness parameter.

## Conclusion

Ultrasound imaging of vena caval diameter fluctuation with respiration is a safe, noninvasive method to assess fluid responsiveness in apneic patients on controlled mechanical ventilation. Two-thirds of patients will not be fluid responsive following an initial volume resuscitation of 30 ml/kg. In spontaneously breathing patients the degree of IVC fluctuation is a function of both respiratory effort and the pressure applied to assist ventilation, and without standardized ventilator settings it has not been proven a reliable indicator of fluid responsiveness.

## Abbreviations

AUROC, area under the receiver operator curve; IVC, inferior vena cava; LVOTO, left ventricular outflow tract obstruction; PLR, passive leg raising; PPV, pulsed pressure variation
